# Brunner's Gland Hyperplasia in a Patient after Roux-Y Gastric Bypass: An Important Pitfall in GLP-1 Receptor Imaging

**DOI:** 10.1155/2020/4510910

**Published:** 2020-04-03

**Authors:** Matthias Hepprich, Kwadwo Antwi, Beatrice Waser, Jean Claude Reubi, Damian Wild, Emanuel R. Christ

**Affiliations:** ^1^Division of Endocrinology, Diabetology and Metabolism, University Hospital Basel, Petersgraben 4, 4053 Basel, Switzerland; ^2^Clinic of Endocrinology, Cantonal Hospital Olten, Basler Strasse 150, 4600 Olten, Switzerland; ^3^Division of Nuclear Medicine, University Hospital Basel, Petersgraben 4, 4053 Basel, Switzerland; ^4^Institute of Pathology, University of Berne, Berne, Switzerland; ^5^Center for Neuroendocrine and Endocrine Tumors, University Hospital Basel, Petersgraben 4, 4053 Basel, Switzerland

## Abstract

Severe cases of postprandial hypoglycaemia after bariatric surgery can be a diagnostic and therapeutic challenge. The diagnostic role of ^68^Ga-DOTA-Exendin-4 PET/CT in postbariatric hypoglycaemia for further treatment decisions is unclear. We present a case of a 50-year-old woman with frequent and severe postprandial hypoglycaemic (≤2.5 mmol/L) episodes starting three years after Roux-Y gastric bypass. Despite strict dietary adherence and several medical therapies, the patient remained severely affected, and ^68^Ga-DOTA-Exendin-4 PET/CT was performed to exclude atypical presentation of an insulinoma or nesidioblastosis. No pancreatic abnormalities were found, but intensive tracer accumulation in the first and second part of the duodenum was detected, which proved to be hyperplastic Brunner's glands on histology and were strongly positive for the glucagon-like peptide-1 receptor. This case provides histopathological verification that duodenal ^68^Ga-DOTA-Exendin-4 uptake is caused by uptake in Brunner's glands and points to a potential relationship between bariatric surgery and Brunner's glands.

## 1. Introduction

Postbariatric hypoglycaemia (PBH), formerly also described as late dumping, is a significant but under-recognized medical complication after bariatric surgery [1, 2]. Depending on the diagnostic test, recent data suggest higher incidence rates than previously thought, occurring in up to 48% of patients after Roux-Y gastric bypass and up to 25% of patients after sleeve gastrectomy [3–5]. The characteristic hallmark of PBH is a rapid increase in blood glucose shortly after ingestion of carbohydrates followed by increased insulin secretion, resulting in postprandial symptomatic hypoglycaemia [1]. The exact mechanisms of PBH have not been fully elucidated, and thus, no approved medical treatment exists so far. Patients, uncontrolled with dietary modifications, are often treated with off-label medications according to individual case-finding strategies [1, 6]. Based on controversial data suggesting an increase in *β*-cell mass or even development of nesidioblastosis, some severely affected patients were reported to be either anatomically reverted or (hemi)pancreatectomized with varying success rates [6].

For patients with other forms of endogenous hyperinsulinaemic hypoglycaemia, such as insulinoma or adult nesidioblastosis [7], glucagon-like peptide-1 receptor (GLP-1R) imaging using ^68^Ga-DOTA-Exendin-4 PET/CT has been reported to be very useful in localizing the insulinomas or focal nesidioblastosis [7, 8]. However, in vitro data using autoradiography in pancreatectomized patients with severe PBH indicate that the density of GLP-1R is not increased in islets compared to normal islets and is significantly lower than that in insulinomas [9]. GLP-1R imaging may, therefore, not be useful in patients with PBH to prove possible nesidioblastosis in PBH. Nevertheless, GLP-1R imaging *in vivo* has not yet been reported in patients with PBH.

## 2. Case Presentation

**Figure 1:**
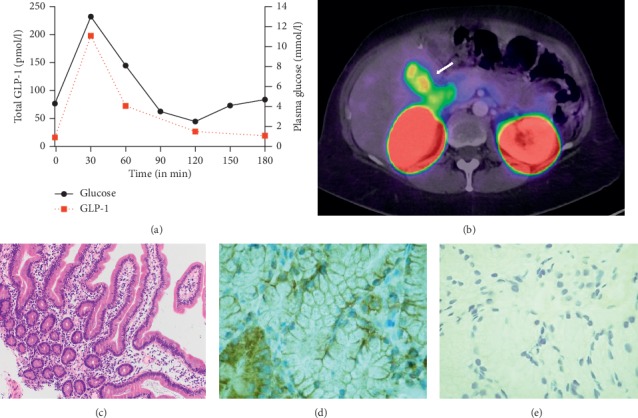
(a) Glucose (black circles) and total GLP-1 (red dashed lines) plasma levels (measured by Mercodia AB, Uppsala, Sweden (assay # 10-1278-01) of the patient during a standardized mixed-meal test 300 ml Ensure plus®, Abbott, containing 60 g carbohydrates). The patient developed symptomatic hypoglycaemia at a timepoint of 120 min (2.5 mmol/l) requiring intravenous glucose administration with immediate resolution of symptoms. (b) Representative ^68^Ga-DOTA-Exendin-4 PET/CT image of the patient with a large and strong tracer accumulation (SUVmax 10) in pars 2 duodenii (white arrow). (c) Haematoxylin and eosin staining of a representative duodenal biopsy at 100x resolution showing hyperplastic Brunner's glands with (d) GLP-1 receptor immunostaining of Brunner's glands (membranous distribution) and (e) negative immunostaining for insulin each at 200x resolution. All staining was performed with a respective positive control (data not shown).

A 50-year-old woman was admitted to our outpatient clinic for medical workup of a severe form of postprandial hypoglycaemia. Seven years earlier, a Roux-Y gastric bypass was performed due to morbid obesity (preoperative BMI 44.7 kg/m^2^and weight 129 kg). Within two years, the patient lost 57 kg (BMI 25.5 kg/m^2^). A year later, the patient noticed, for the first time, hypoglycaemic episodes 1-2 hours after meal intake, which were mainly characterized by loss of concentration, severe fatigue, and auditory and visual impairment that quickly resolved after intake of carbohydrates. An oral glucose tolerance test, at that time, confirmed the presence of symptomatic postprandial hypoglycaemia (2.9 mmol/l). Symptoms persisted despite dietary modifications of up to twelve small meals, each containing a maximum of 20 g of carbohydrates. Off-label treatment with acarbose, saxagliptin, and metformin by her treating physician reduced the frequency and severity of hypoglycaemic episodes but did not significantly improve the situation. Eventually, the patient was referred to our hospital. Her remaining personal history included a mild form of orthostatic dysregulation, migraine, and a multilocular nodular hepatic hyperplasia that required a left-sided hemihepatectomy 17 years ago. The clinical examination was unremarkable apart from irritation-free scars after hemihepatectomy (height, 168 cm; weight, 72 kg; BMI, 25.5 kg/m^2^; blood pressure, 127/67 mmHg; regular heartbeat, 80 bpm). Laboratory analysis including blood smear; biochemistry comprising electrolytes and kidney and liver parameters; glycated haemoglobin; blood lipids; iron; vitamins B1, B6, B12, and D; and zinc were within reference ranges. A continuous glucose flash monitoring system confirmed a typical pattern of postbariatric hypoglycaemia without any signs of fasting or nocturnal hypoglycaemia. A mixed-meal test (300 ml Ensure plus®) containing 60 g of carbohydrates after a 10-hour fasting period confirmed symptomatic postprandial hypoglycaemia (sweating, drowsiness, odd behaviour, and incoordination) at a glucose level of 2.5 mmol/l, and intravenous glucose administration was required for immediate remission of symptoms (Figure 1(a)). Due to the severe symptomatic presentation of the patient, a ^68^Ga-DOTA-Exendin-4 PET/CT was performed to exclude an atypical presentation of an insulinoma or focal nesidioblastosis, which might be a surgical target [10–12]. Therein, the pancreas showed a homogeneous signal distribution (SUV 5.7–8.3), but intensive uptake in the first and second part of the duodenum (SUV_max_ of 10.0) was observed (Figure 1(b)). To further differentiate this unexpectedly strong tracer accumulation, a double-balloon enteroscopy was performed, which revealed macroscopically unremarkable intraluminal structures in the duodenum. Histologically, there were no signs of malignancy or inflammation (Figure 1(c)). Representative biopsies of the pars 2 duodenii showed normal mucosa with hyperplastic Brunner's glands, which were strongly positive for the GLP-1 receptor on immunohistochemistry but negative for insulin (Figures 1(d) and 1(e)).

## 3. Discussion

This is the first case of PBH with a histologically proven GLP-1R-mediated increase in ^68^Ga-DOTA-Exendin-4 uptake in hyperplastic Brunner's glands of the duodenum.

A complex interplay of several factors is important for the development of PBH rather than a dominant single factor [1]. One pivotal factor is the glucagon-like peptide-1 (GLP-1) concentration, which is significantly increased in patients after bariatric surgery and specifically in those patients with PBH [2, 13, 14]. Antagonizing its effect by using exendin 9–39 lowers the rate of PBH [15]. However, there are also reports of successful treatment with GLP-1 agonists [16], but data on its mechanisms still need to be unravelled [17]. There is currently no commercially and medically approved compound available for the treatment of PBH [1]. On the other hand, GLP-1R imaging is a new molecular imaging method that is useful for the localization of insulinomas and eventually adult nesidioblastosis in patients with endogenous hyperinsulinaemic hypoglycaemia (EHH) [7, 8]. The physiological background relies on high expression levels of GLP-1R in benign insulinomas and adult nesidioblastosis. Studies using autoradiography indicate that normal pancreatic islets express approximately six times less GLP-1R than insulinomas [9, 18, 19].

The hyperplasia of Brunner's glands and high accumulation of ^68^Ga-DOTA-Exendin-4 in those Brunner's glands resulting in a positive GLP-1R PET/CT scan (Figure 1(b)) is a surprising finding. The underlying pathophysiological mechanism is unclear. Brunner's glands are mucinous glands that are primarily located in the proximal duodenum, where their glycoprotein-rich mucus serves as a protective barrier for underlying structures against acidic and pepsin-containing agents from the stomach. Incretins (glucagon, GLP-1, vasoactive intestinal peptide, secretin, and cholecystokinin) and neuronal factors (acetylcholine) stimulate mucus and bicarbonate secretion from the glands [20]. These glands have been convincingly shown *in vitro* to express GLP-1R in high density and with a strong membranous pattern via immunohistochemistry staining, reflecting the localization of this G-protein coupled receptor, as depicted in Figure 1(d) [19, 21]. Duodenal GLP-1R appears to be important for neuro-glucoregulation and gut-lipid-sensing [22]. Recent data also suggest a role for pathogen defence, mucosal layer protection, and mucosal healing by targeting GLP-1R in Brunner's glands [23]. Whether these murine data translate to humans and whether ligands other than GLP-1 play a role remain uncertain [24]. It is conceivable that in patients with PBH, increased GLP-1 levels but also other incretins such as GLP-2 with trophic effects may also lead to hyperplasia of Brunner's glands, as observed in this patient [25, 26]. However, the distinct pathophysiological role and the interrelationship between GLP1, GLP-1 receptors, and hyperplasia of Brunner's glands remain to be determined.

Christ et al. speculated in 2009 that Brunner's glands may explain the false-positive result in GLP-1 receptor imaging due to the close location of a lesion to the pancreatic head [27]. To the best of our knowledge, this is the first histologically proven GLP-1R-mediated uptake of ^68^Ga-DOTA-Exendin-4 in duodenal Brunner's gland hyperplasia detected with PET/CT (Figure 1). More importantly, immunohistochemistry of Brunner's glands was negative for insulin (Figure 1(e)), suggesting that hyperplasia of Brunner's glands is not related to autonomous insulin secretion in this patient. However, the relatively high GLP-1 levels observed in this patient compared to other published data [14] may be related to the clinical presentation and/or the hyperplasia of Brunner's glands. Generally, hyperplasia of Brunner's glands is reported to occur in response to an acidic environment or *Helicobacter pylori* infection and can even be obstructive [28]. However, in this patient, the remainder of the histological examination did not find any signs of inflammation or *H. pylori* colonization. The exact factor leading to Brunner's gland hyperplasia remains to be determined. In addition, whether these changes are critical for the occurrence of postprandial hypoglycaemia after bypass surgery needs to be evaluated.

## 4. Conclusions

This case indicates a potential relationship between gastric bypass surgery, PBH, GLP-1, GLP-1R, and Brunner's glands. Further investigation is warranted to identify the role of GLP-1 receptors in Brunner's glands in patients after bariatric surgery and specifically in those affected by postbariatric hypoglycaemia.

GLP-1 receptor-positive Brunner's glands are an important differential diagnosis of positive ^68^Ga-DOTA-Exendin-4 PET/CT and a possible pitfall for the location of insulinomas.
